# An Insight into the Correlation between Chemical Composition Changes of Aluminum-Iron-Polyphosphate Glasses and Thermal Properties

**DOI:** 10.3390/ma14082065

**Published:** 2021-04-20

**Authors:** Pawel Goj, Aleksandra Wajda, Agata Stoch, Ireneusz Krakowiak, Pawel Stoch

**Affiliations:** 1 Faculty of Materials Science and Ceramics, AGH University of Science and Technology, 30-059 Krakow, Poland; pgoj@agh.edu.pl (P.G.); pstoch@agh.edu.pl (P.S.); 2 The Łukasiewicz Research Network, Institute of Electron Technology Krakow Division, 30-701 Kraków, Poland; agata.stoch@gmail.com; 3 Faculty of Automotive and Construction Machinery Engineering, Warsaw University of Technology, 02-524 Warsaw, Poland; ireneusz.krakowiak@pw.edu.pl

**Keywords:** aluminum-iron-phosphate glass, thermal properties, thermal analysis, glass network

## Abstract

The present study aimed to investigate the influence of the gradual substitution of Fe_2_O_3_ by Al_2_O_3_ on the thermal properties of polyphosphate glasses. The conducted considerations based on differential scanning calorimetry (DSC) and heating microscopy thermal analysis provided much essential information about the correlation between glass chemical composition and its characteristic parameters, such as transformation temperature, specific heat, crystallization temperature, crystallization enthalpy, the activation energy of crystal growth, melting temperature, and Angell glass thermal stability. The obtained estimation of viscosity changes as a function of temperature could be very helpful for researchers to correctly plan the vitrification process and thus radioactive waste immobilization. A precise analysis of DSC curves and X-ray diffraction patterns revealed the possibility of crystallization process design in order to create materials with different levels of crystallinity and phase composition. The drawn conclusions allow choosing the glass with the optimal concentration of Al_2_O_3_ and Fe_2_O_3_, which ensures the relatively low melting temperature, viscosity, and glass crystallization ability, with application potential in nuclear waste immobilization.

## 1. Introduction

Phosphate glasses are important materials due to their versatile applications as well as scientific interest. Their chemistry differs significantly compared to traditional silicate glasses, which is related to the different crystallochemical properties of phosphorus, which is the main network element, over silicon [1]. Pure or high phosphorus content glasses are characterized by relatively low chemical durability, which is the effect of the abundance of easily hydrated P-O-P bonds. This phenomenon strongly limits their potential applications. Nevertheless, the partial substitution of phosphorus by iron or aluminum greatly improves the chemical durability of the glasses, which results in the possibility to obtain the material with superior leaching properties. This, among others, makes them a good candidate to be used in waste vitrification technology [2,3].

The constant development of the nuclear industry causes the necessity of the safe disposal of radioactive by-products. The waste due to containing radionuclides has to be isolated from the environment for a very long period, reaching the geological scale. Therefore, it is necessary to search for new methods and materials in which the waste may be safely immobilized. One of the promising methods of waste stabilization is closing them in the flexible structure of glasses [2,3]. The application of iron-phosphate glasses (IPG) has been extensively studied in the field of high-level radioactive waste (HLW) immobilization [4]. The large loading capacity of waste rich in Cs, Mo, U, Cr, La, Hf, Ce, Pu is possible due to the advantages of thermal properties of phosphate glasses (low glass transition temperature and low melting temperature), ensuring the high dissolution rate of rare earth and heavy metals. Additionally, the relatively low viscosity of the melt results in a decrease in homogenization time, which limits the evaporation of volatile components. Thus, the secondary waste from off-gas systems and costs of the process are limited [4,5,6,7]. The presence of P-O-Fe bonds in IPG structure guarantee improved chemical durability compared to pure phosphate glasses, which makes IPG a good candidate for HLW vitrification, providing long-term environmental protection [5].

Among the new glass compositions, aluminum-iron-phosphate glasses (AIPG) seem to be a more promising group of materials due to the higher resistance to devitrification and chemical durability. The addition of aluminum can significantly change the thermal properties of the glass depending on its composition. The creation of P-O-Al may increase the glass structure and microstructure homogenization [8,9], mechanical strength, and the solubility of rare-earth ions [10,11].

Both aluminum and iron ions can play an intermediate role in the phosphate glass network. It means that they can be network modifiers leading to network depolymerization or, under specific conditions, may act as network formers [12]. Depending on the glass composition, aluminum and iron could take different coordination numbers, which, in consequence, determine different glass properties. The thermal analysis and their correlation with systematic changes in the proportions of the chemical composition may appear as interesting relationships, which could be helpful in designing future glass for waste immobilization [13].

Glasses for waste vitrification should have a high glass-forming ability (GFA) and high glass thermal stability (GS). GFA determines how easily an amorphous material can be obtained from the melt without crystallization, and it is associated with a slow crystallization rate. Therefore, glass characterized by high GFA can be cooled down at a slower rate. This is important in the case of vitrification, while the melt is frequently poured into large barrels where it is cooled down relatively slowly. The GS parameter is the resistance of glass against crystallization during reheating and can be expressed by the Angell parameter (K_A_), which is the difference between the glass transformation and crystallization temperature [14,15]. Glasses for waste immobilization should also have low melting temperatures below 1200 °C to reduce the evaporation of volatile components like Cs and Ru [2]. The low melting temperature also reduces costs and generation of the secondary waste from the off-gas system. Additionally, the evaporation can be limited by the low viscosity of the melt, which may reduce the melt homogenization time. On the other hand, the low melt viscosity at the processing temperature may be an indicator of rather low GFA. In this way, the optimal glass composition, which is the outcome of the high GFA and low melt viscosity, is highly desirable.

Thermal methods, such as differential scanning calorimetry (DSC), are widely applied for the characterization of the glasses and glass melts [15,16,17,18,19]. It allows us to determine the characteristic glass temperatures, e.g., transformation, crystallization, liquidus temperature, GS parameters, as well as thermodynamic properties [17]. DSC can be also used to estimate the viscosity−temperature curve. This curve is greatly important for industrial glass production because the viscosity changes are an essential parameter for every step during glass production, e.g., melting, fining, forming, and annealing. Besides, it is also helpful for understanding crystallization kinetics, glass formation, and glass transition. Direct measurement of the viscosity of glasses is rather difficult, and several different measurement techniques must be applied [16,20]. Nevertheless, it has been shown that from the single DSC measurement, the viscosity can be obtained for the entire technological relevant temperature range [1,21].

Taking all the above into consideration, the subject of the study is polyphosphate glasses of the composition 30Fe_2_O_3_-70P_2_O_5_, in which the Fe_2_O_3_ is gradually substituted by Al_2_O_3_. The composition of the base glasses is close to the optimal 40Fe_2_O_3_-60P_2_O_5_ from a waste vitrification point of view. The lower quantity of Fe_2_O_3_ ensures the possibility to increase it by introducing the intermediate oxides from the waste. The main goal of the study was to test the influence of the substitution on the thermal properties of the glasses and to check the possibility of obtaining the glass-ceramic materials. In this way, the usefulness of the glasses in waste processing, from thermal properties perspective and the optimal glass composition, is going to be determined.

## 2. Materials and Methods

Polyphosphate glasses of general formulae 70P_2_O_5_-(30-x)Fe_2_O_3_-xAl_2_O_3_ (mol%) were synthesized using a conventional glass melting and quenching method. In the studied system, Fe_2_O_3_ was gradually substituted by Al_2_O_3_ with x = 5, 10, 15, 20, 25, 30 (see Table 1). The batches were prepared by careful homogenization in a planetary ball mill using appropriate amounts of chemical purity NH_4_H_2_PO_4_, Al_2_O_3_, and Fe_2_O_3_. The mixtures were melted in an electric laboratory furnace in Al_2_O_3_ crucibles. The melting temperature was 1200 °C for the samples, with x from 0 to 20; for the higher Al_2_O_3_ content glasses, the temperature was raised by 100 °C. The melt was vitrified by casting onto a steel plate. During melting at temperatures above 1300 °C, there was evidenced evaporation of P_2_O_5_ [22]. To compensate for P_2_O_5_ losses, an approximately 20% overweight of NH_4_H_2_PO_4_ was used [12].

The chemical composition of the obtained glasses was checked by X-ray fluorescence spectroscopy (XRF) and was consistent with the assumption of the experimental uncertainty limit. Samples for XRF were prepared by pressing glass powders into thin tablets. The investigation was carried out using Axios mAX WDXRF X-ray fluorescence spectrometer with Rh lamp of power 4 kW (PANalytical, Malvern, UK). The analysis was carried out using a standardless method. The uncertainty of measurement was about 5%. The chemical composition and the sample designation are presented in Table 1.

Heating microscopy thermal analysis was carried out using compacted powder samples of a cubic shape. Powdered samples prepared by milling of bulk samples in a ball mill were wetted in ethanol and compacted to cubes of 3 × 3 × 3 mm^3^ by a hand press. The shape changes during heating were observed by the Carls Zeiss MH01 microscope (Carls Zeiss, Jena, Germany) at the heating rate of 10 °C min^−1^. Data of the sample height were collected at intervals of 10 °C during the experiment, and shrinkage curves were obtained. The beginning of the sintering process temperature (T_s_) as the onset of the densification on the shrinkage curve was designated. The full sintering temperature T_FS_ was defined as the point where the densification is stopped, and the shape of the sample is the same as at the T_s_ point. The temperature when the sample takes a spherical shape is designated as T_SPH_. The flow temperature Tf was defined as the first temperature at which the sample is melted to a third of its original height [23]. Additionally, the dependence of shrinkage (h/h_0_–the ratio of a sample height at a given temperature to the height at room temperature) on temperature was derived.

The powdered glass samples were the subject of differential scanning calorimetry combined with thermogravimetry (TG). Measurements were carried out using Netzch STA449 F5 Jupiter (Netzch, Selb, Germany), operating in the heat flux DSC mode. The samples weighing 80 mg were heated in Al_2_O_3_ crucibles at a rate of 10 °C min^−1^ in a dry air atmosphere up to 1100 °C. Based on the measured curves, the following characteristic temperatures and effects were determined: mid-point of the transformation step (T_g_), accompanying change in the specific heat (∆Cp), onset (T_g_′) and the end-point of the step (T_g_″), the onset of crystallization peak (T_C_), crystallization enthalpy (∆H), the maximum endothermic effect of the high temperature (T_M_). The parameters were estimated using the Netzsch Proteus Thermal Analysis Program (version 5.0.0., Netzsch, Selb, Germany). The activation energy of the crystal growth (E_C_) was estimated based on the differential DSC curve, as described in [24,25]. The method gives an easy way to roughly estimate the activation energy based on the position and the width of the crystallization effect on the DSC curve.

The synthesized glasses were the subject of devitrification. The process was conducted in an electric laboratory furnace at the crystallization temperature obtained from the DSC measurements. The powdered glass samples were kept at that temperature for 48 h in the air atmosphere. The crystallized glass samples were tested using X-ray diffractography. The measurements were carried out by PANalytical X’Pert Pro diffractometer (PANalytical, Malvern, UK) and Cu K_α1_ radiation. The analysis of the obtained XRD patterns was conducted using PANalytical X’Pert Pro software (version 3.0.4, PANalytical, Malvern, UK). The crystallizing compounds were identified based on the data summarized in Crystallography Open Database [26].

## 3. Results and Discussion

**Figure 1:**
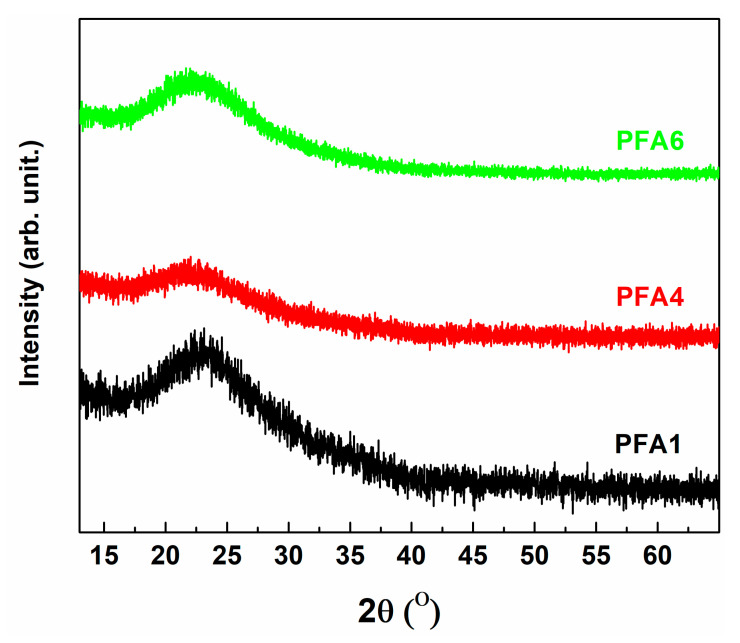
The confirmation of the amorphous nature of obtained glasses using XRD analysis.

The amorphous nature of the investigated materials is confirmed by X-ray diffraction. In Figure 1, three exemplary diffractograms without any reflections are presented. One broad characteristic amorphous ‘halo’ in XRD patterns is clearly visible. The absence of any additional ‘halos’ is proof that the liquation phenomenon does not occur. In the case of glass phase separation, two adjoined large maxima should be noticeable, as presented in previous studies [27,28,29,30].

**Figure 2:**
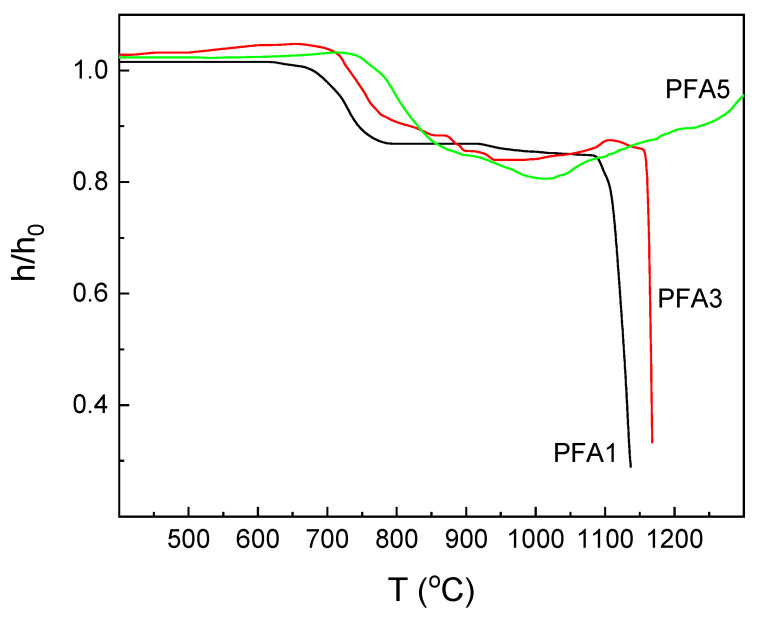
Dependence of the glass shrinkage on temperature for the PFA1, PFA3, and PFA5 samples.

**Figure 3:**
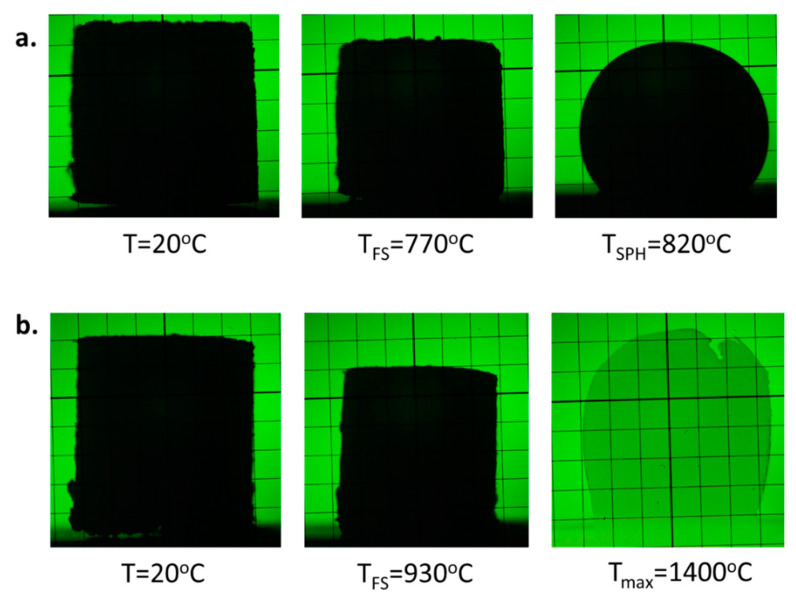
Examples of glass images at the selected temperatures for glasses: (**a**) PFA1, (**b**) PFA6.

The heating-stage microscopy is a quite convenient tool to describe the basic thermal properties of glasses. Observation of the sample shape changes during heating can bring important information, especially from a technological point of view. The observations are conducted for all the obtained materials. Depending on the Al_2_O_3_ content in the glass composition, three different types of shrinkage curves of the samples are obtained. The first one is for the glasses with the lowest Al_2_O_3_ content (PFA1, PFA2), and the second is for the samples with the middle content (PFA3, PFA4). The third one is for the glasses with the highest Al_2_O_3_ concentration (PFA5, PFA6). The exemplary curves for the selected ranges are presented in Figure 2, and examples of glass images at the selected temperatures for glasses PFA1 and PFA2 are shown in Figure 3. Based on the obtained curves and the images, the characteristic temperatures are determined and are summarized in Table 2.

The course of the shrinkage–temperature curves of the glasses with the lowest Al_2_O_3_ content behaves in a typical way like for most silicate glasses (Figure 2, curve PFA1). In the beginning, a slight increase in the sample size with the temperature is observed, which is related to the thermal expansion of glass grains during heating. Then, a considerable shrinkage is observed, which starts at T_S_ temperature, and the samples are densified while maintaining their shape. Finally, further shrinkage is stopped, and the full sintering is obtained (Figure 3a, T_FS_). Further, an increase in the temperature results in a gradual decrease in the viscosity of the material, which is observed as rounding of the sample corners and the beginning of the softening of the sample. The sample starts to take a spherical shape (Figure 3b, T_SPH_). With the increase in the temperature, the viscosity of the material is going down, and there is a rapid drop in the glass melting curve until flow temperature T_f_ is achieved.

The glasses with middle Al_2_O_3_ content behave similarly as above (Figure 2, curve PFA3). The difference is that above T_SPH_ temperature, small changes in the shapes of the samples are detected. It indicates an effect of partial crystallization of the sample and formation of the glass-ceramic material. Although the viscosity of the vitreous phase is decreased with the temperature, the expansion of the samples while maintaining their shape and maximum characteristic temperature at about 1100 °C and 1200 °C for PFA3 and PFA4 samples, respectively, is observed. At higher temperatures, slow shrinkage can be noticed. It may be related to the melting of the glassy phase, whereas the shape is still held by the crystalline phase. Finally, the shrinkage becomes rapid, which may be the result of the final melting of the crystalline phase.

There is neither above maximum nor the melting point in the considered temperature range for the glasses with the high alumina content (Figure 2, curve PFA5; Figure 3b, T_max_). This is due to the possible glass devitrification with the formation of the high-temperature melting crystalline phases, e.g., AlPO_4_.

**Figure 4:**
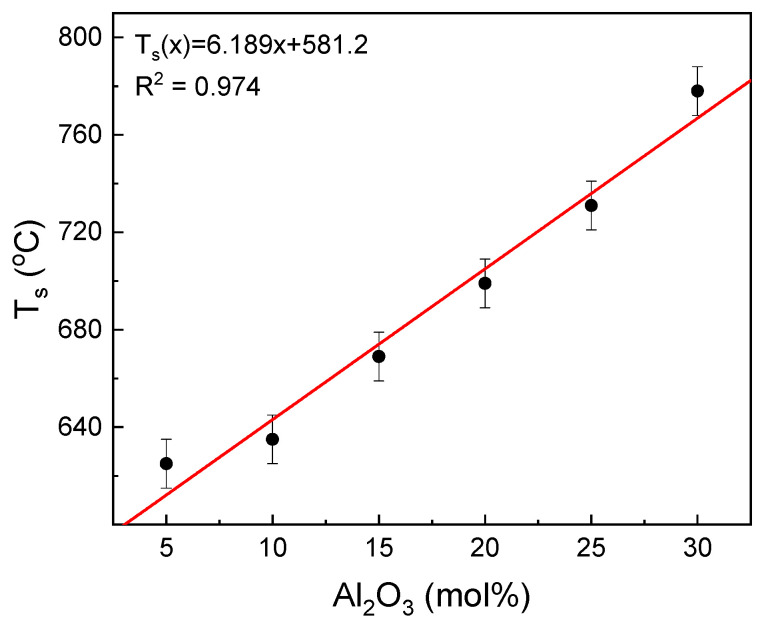
Dependence of T_S_ temperature on the glass composition.

The influence of the glass composition on the glass sintering temperature is shown in Figure 4.

The increase in the aluminum content in the glass leads to a linear increase in the sintering temperature of the glass. The increase is in accordance with the following formulae: T_s_(x) = 6.189x + 581.2 (°C), where x is the mol% of Al_2_O_3_ in the tested glasses. Thus, the 1 mol% substitution of Fe_2_O_3_/Al_2_O_3_ raises the temperature by about 6 °C. Taking this into account, the glass sintering starts at the point where the glass viscosity log(η) is in the range of about 9.0 to 10.0 [31]. This suggests that the substitution increases the glass viscosity at the T_s_ temperature.

**Figure 5:**
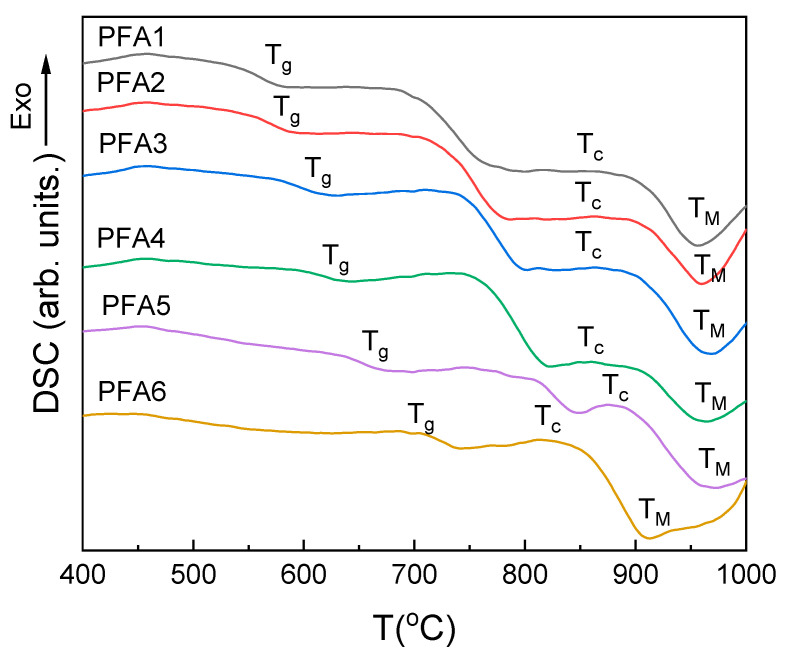
DSC curves of the studied glasses.

The DSC curves of the studied glasses are presented in Figure 5, and the obtained characteristic temperatures and properties, such as T_g_, T_g_′, T_g_″, ∆C_p_, ∆H, E_C_, T_c_, and Angell thermal stability parameter (K_A_ = T_c_ − T_g_), are summarized in Table 3.

All the investigated glasses show characteristic glass transformation steps on the DSC curves (Figure 5, T_g_–point). After the step, the curves become flat, and with the increasing temperature, another step is evidenced whose position and range overlap with the maximum densification on the shrinkage curves (Figure 2) and may be related to softening of the glass. After the step, there is no decrease, and the weak and broad exothermic effects related to glass crystallization are evidenced (Figure 5, T_c_–point). The broad character of the effects suggests that the crystallization has a surface nucleated character, and their kinetics are relatively slow. Just after the effects, a further drop in the DSC curves is observed due to a gradual decrease in the glass viscosity. Finally, the minimum decrease is achieved. The effect can be interpreted as the final dissolution of the glass grains or crystals (Figure 5, T_M_–point). The broad character of the endothermic effect does not suggest that due to the melting of the crystalline compounds in the considered temperature range. Additionally, the idea of the glass grains dissolution is supported by the observation of the rapid decrease in the corresponding shrinkage curves (Figure 2). Thus, above the point, the glass should be considered as a continuous liquid [32].

**Figure 6:**
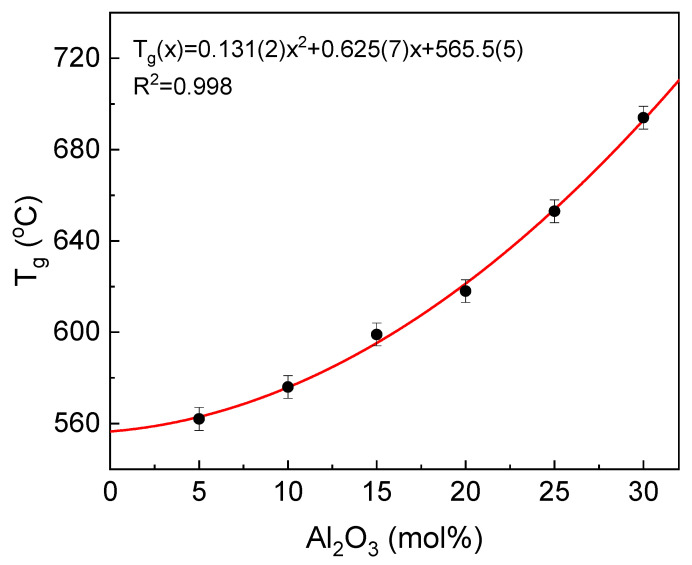
Dependence of T_g_ temperature on glass composition.

One of the most important technological as well as scientific parameters describing glasses is glass transition temperature (T_g_). This is the temperature where glass in a solid form is transformed to a liquid state [33]. The compositional dependence of the temperature is presented in Figure 6.

It can be seen that the T_g_ temperature increases with the aluminum concentration of the glass. The increase is in accordance with the formulae T_g_(x) = 0.131(2)x^2^ + 0.625(7)x + 565.5(5) (°C), where x is the mol% of Al_2_O_3_ in the glass. The increase in the T_g_ temperature is related to the increase in the glass network stiffness, as defined and discussed in [34,35]. The network becomes more rigid, and in this way, more energy needs to be provided to start the atom reorganization and to activate the rotational motions of atoms [23,35,36,37]. According to our previous studies concerning the influence of aluminum on structural features of the IPG, we demonstrate that aluminum increases considerably the glass network polymerization. The effect is achieved by the higher aluminum preference in the occupation of tetrahedral network position in comparison to ferric iron. Additionally, part of iron atoms is reduced to ferrous (about 20–30%). In this redox state, iron acts as the glass network modifier, making the network more flexible [12]. Moreover, the ionicity of the Fe^2+^-O bonds is higher than Fe^3+^-O and Al^3+^-O, which have, among them, the most covalent character [38]. The covalent bonds are more rigid compared to ionic. Thus, an increase in the glass network polymerization induced by Fe_2_O_3_/Al_2_O_3_ substitution together with the increase in mean covalency of the bonds leads to the higher glass network rigidity and a rise in the transformation temperature.

**Figure 7:**
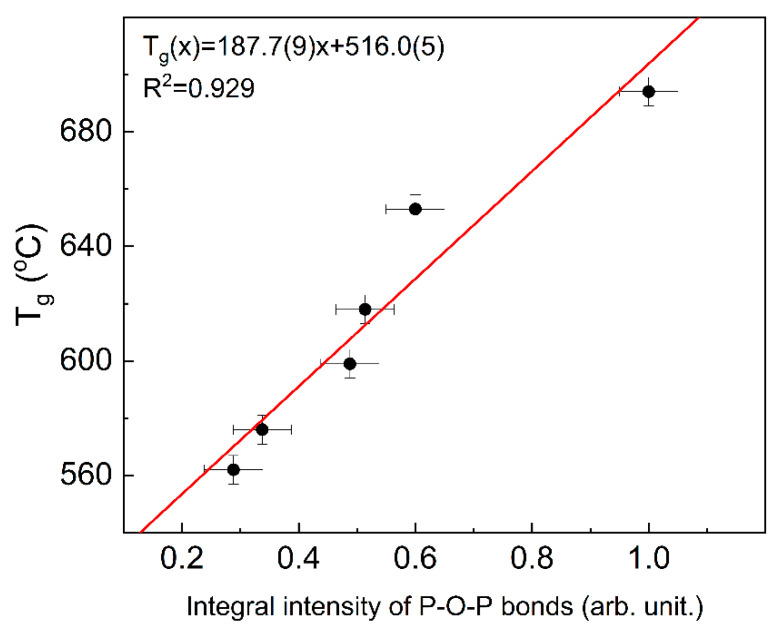
Dependence of T_g_ temperature on Raman integral intensity of P-O-P bonds (the integral intensities from [12]) (line for eye guides only).

The indicator of the glass network polymerization may be the number of P-O-P bridges, which is proportional to the integral intensity of the appropriate band in Raman spectroscopy. Based on the data presented in [12], a correlation between the integral intensity and T_g_ temperature can be observed. The correlation is presented in Figure 7.

It can be seen that the T_g_ temperatures follow linearly the P-O-P integral intensity and thus increase with the glass network polymerization. Additionally, it shows that in this case, the network polymerization increase is predominant over the change in the chemical bond character. This also shows that changes in T_g_ temperature can be treated as an indicator of the glass network polymerization degree.

**Figure 8:**
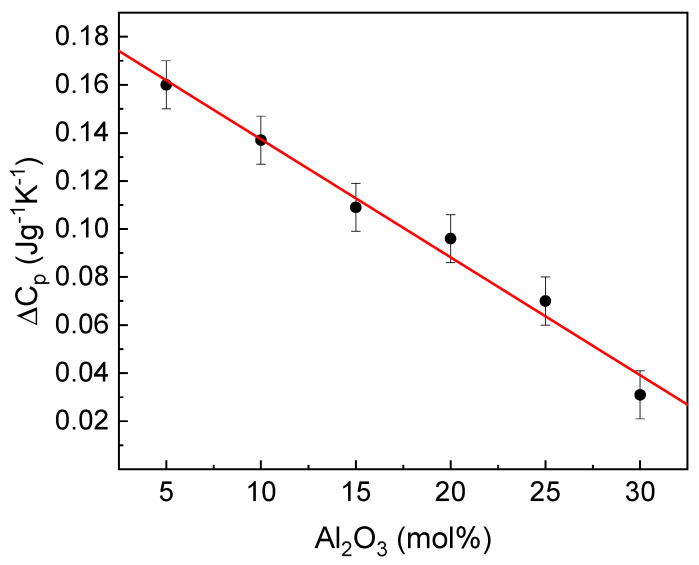
Dependence of ΔC_p_ on the glass composition (line for eye guides only).

Another useful parameter related to glass transition is the change in the heat capacity accompanying the transition. The dependence of the parameter on glass composition is presented in Figure 8.

The Fe_2_O_3_/Al_2_O_3_ substitution linearly decreases the change in the heat capacity. The parameter can be considered as an indicator of a degree of the structural changes accompanying the transformation, like the number and strength of the broken bonds and rearrangement of structure constituents, which affect the value of the configuration entropy. This comprises the number and the energy of the broken chemical bonds. Thus, the parameter change is related to the differences in the bond’s ionicity, the lower ionicity, the lower value of ∆Cp [12,39]. As was mentioned above, the increase in Al_2_O_3_ enhances the mean covalent character of the cation-oxygen bonds, and as a result, the network becomes more rigid. Consequently, it should cause an increase in T_g_ [12], as observed in this case.

**Figure 9:**
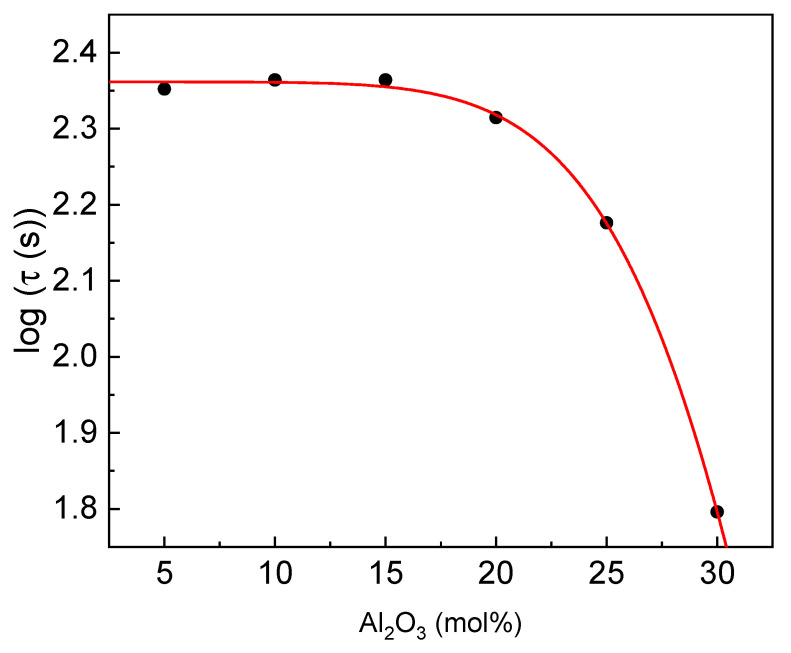
Log total relaxation time τ versus Al_2_O_3_ content (line for eye guides only).

The glass transition during which the sample transforms into a supercooled liquid state is a process of diffusion. The more time the glass transition needs, the higher is the glass-forming ability. Therefore, the total relaxation time for the glass transition (τ) can be approximated as τ = ΔT_g/_β, where ΔT_g_ is the width of the glass transition region (ΔT_g_=T_g_″-T_g_′), and β is the heating rate [40,41,42]. The estimated relaxation time as a function of glass composition is presented in Figure 9.

It is observed that the τ value is almost constant for Al_2_O_3_ concentration up to 15 mol% and then rapidly decreases. Compared to the results with other phosphate glasses [37], it should be concluded that the GFA is higher or similar for the compositions up to 25% of Al_2_O_3_. Therefore, the ability is high, whereas, in the case of the pure aluminum-phosphate glass, the ability is rather moderate.

**Figure 10:**
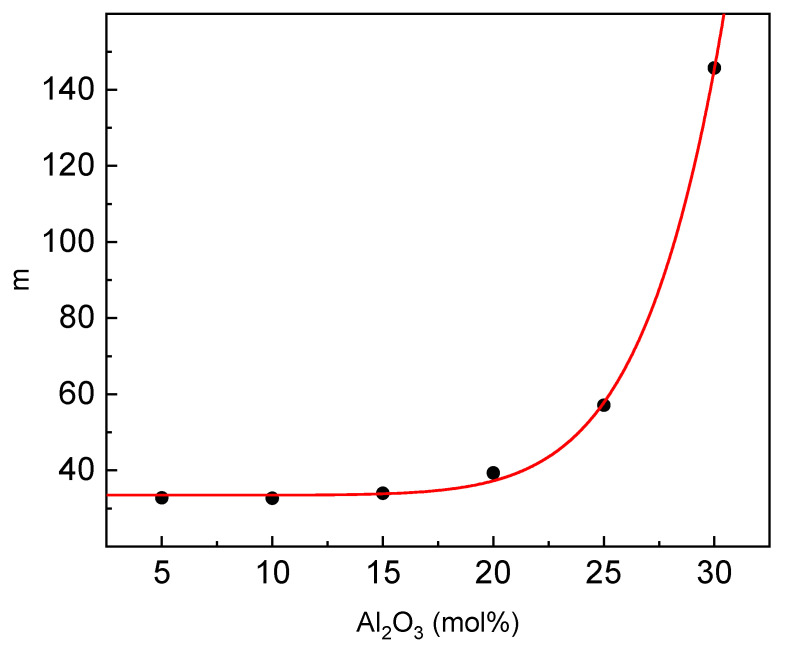
Dependence of fragility parameter m on the glass composition.

Another important parameter describing the GFA is fragility (m). To evaluate the parameter, one can use the approximate formulae m ≈ 2.1T_g_/ΔT_g_ [43]. The estimated dependence of m on the glass composition is given in Figure 10.

The fragility behaves in a similar way to the estimated relaxation time. At low aluminum contents up to 15 mol%, the fragility is almost constant and then rapidly increases. According to Vilgis [44], glass-forming liquids with a low value of m c.a. 16 are characterized as strong glass-forming liquids, whereas those with a high value of m c.a. 200 are characterized as fragile glass-forming liquids. Based on this criterion, the studied glasses, especially of low aluminum content, belong to the strong glass-forming liquids [41].

The next important parameter that can be roughly estimated based on the width of the glass transition step is the temperature dependence of melt viscosity (η). The parameter can be evaluated based on T_g_′ and T_g_″ values according to the model proposed in [1,21]. In this model, the viscosity of the glass-forming melt can be estimated from the following equation:

(1) $$log\eta \left(Pa\cdot s\right)\;\approx \;-5+\;\frac{14.2}{(0.147(T-{T_{g}}^{’’})/{T_{g}}^{’’2}(\frac{1}{{T_{g}}^{’’}}-\frac{1}{{T_{g}}^{’}}))+\;1}$$

**Figure 11:**
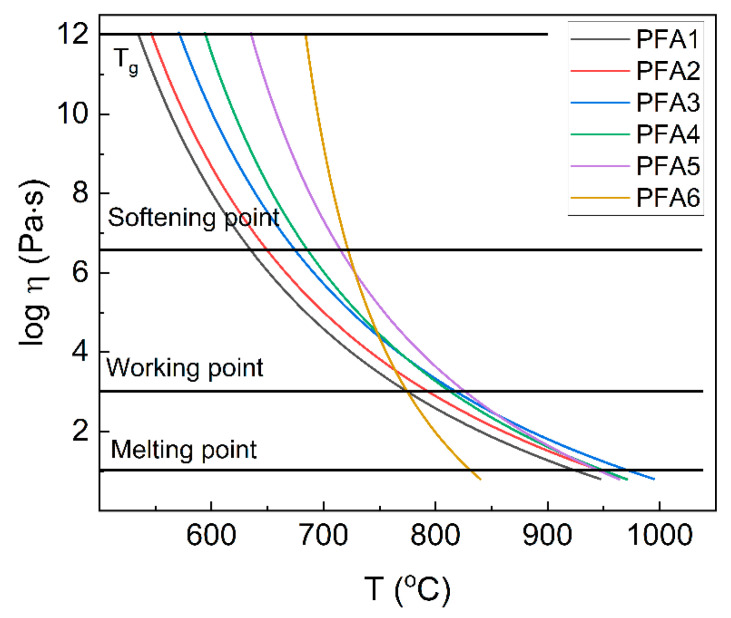
The dependence of estimated viscosity on temperature and important technological points.

The dependence of estimated viscosity on temperature is presented in Figure 11. Based on the viscosity values, important technological points are marked in the figure [1,33].

The glass melting point is defined here as the temperatures, where log η = 1 and the glass is fluid enough to be considered a liquid [32]. The value of the point increases from 926 °C to 975 °C for glasses with the Al_2_O_3_ content up to 15 mol%. The further increase in Al_2_O_3_ leads to a decrease in the temperature to 832 °C for the pure aluminum-phosphate glass. It should be pointed out that the values correspond very well to the experimentally-derived T_M_ values (Table 3). When we compared the viscosity results with the characteristic temperatures determined with the hot-stage microscopy (Table 1), it can be seen that the glass sintering process is finished, and the maximum of the densification is achieved for the temperatures around the glass softening point. Further increase in the temperature leads to a decrease in the viscosity, and the sample starts to take a round shape. Thus, it confirms that although the model is only a rough estimation, it can be used to predict the important technological points and ranges correctly. Based on the data presented in Figure 11, it can be observed that the Al_2_O_3_ content up to 15 mol% increases the melt viscosity, and the glass working range makes the glass “longer”. A similar phenomenon is known for silicate glasses. For the higher aluminum contents, the viscosity quickly decreases, which is explained by an increase in glass network stiffness. The higher the stiffness is, the more energy is needed for the atoms’ reorganization. On the other hand, there is an increase in the mean metal-oxygen covalency. The more the covalent bonds are less flexible, the more fragile they are. Thus, instead of changing the direction, they start breaking, which leads to the decomposition of the network and a sudden decrease in the viscosity. It should be pointed out that more studies should be performed to explain the problem.

**Figure 12:**
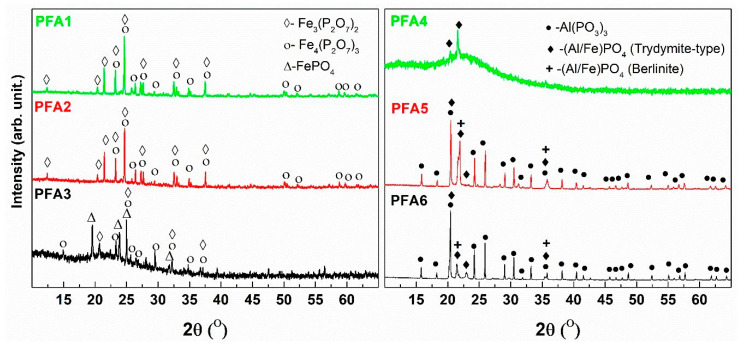
XRD analysis of the devitrified glasses.

At around 850 °C, a weak effect of crystallization is observed, and at this temperature, the glassy samples are subject to devitrification. The results of the XRD analysis of the devitrified materials are presented in Figure 12.

The XRD patterns of the devitrified materials with low aluminum content (PFA1, PFA2) are characterized by strong and well-developed peaks and low and flat backgrounds, which indicates the high level of crystallinity of the samples. In these cases, two main crystalline compounds are detected, which are mixed iron valency Fe^2+^Fe^3+^_2_(P_2_O_7_)_2_ and ferric Fe_4_(P_2_O_7_)_3_ phosphates. The formation of the mixed-valence compound suggests the partial reduction of the iron in the pristine glass. On the other side, the crystal structure of the ferric phase shows similarities to the mixed-valence structure [45]. Thus, it implies the mixed compound oxidation effect, as proposed in [46]. An increase in the aluminum content leads to the observation that the background of the XRD patterns becomes more intense with a characteristic amorphous halo at about 2Θ = 22°. This indicates a much higher content of amorphous phase in the mid Al_2_O_3_ content samples (PFA3, PFA4). In the case of the PFA3 devitrified material, the same crystalline compound, as for the lower aluminum content samples, is detected. Nevertheless, the formation of rodolicoite (FePO_4_), which is ferric iron-phosphate of the same crystal structure as AlPO_4_ (berlinite), is observed. It should be noted that in the sample, there is an equal content of aluminum and iron, and no pure aluminum-phosphate compounds are detected. Nevertheless, taking into account the crystallochemical similarities of Al^3+^ and Fe^3+^ cations, we suppose that there is a partial substitution of Fe by Al in the iron-phosphate compounds. In the case of the higher Al_2_O_3_ content in the PFA4 devitrified sample, we did not detect iron-phosphate compounds, and only weak peaks characteristic for AlPO_4_ in the tridymite-type structure were evidenced. The character of the pattern indicates the lowest level of the crystallinity of the material. The observation suggests that the increase in the aluminum content prevents the crystallization of the iron-phosphate compounds. On the other hand, for the glasses with the higher aluminum content, the patterns suggest the higher crystallinity level of the materials. In these cases, iron cations prevent crystallization. Thus, crystallization of aluminum-phosphate compounds without the typical iron-phosphates can be observed. The main crystalline compounds are AlPO_4_ in both polymorphic forms (berlinite, tridymite) and Al(PO_3_)_3_. The detailed studies of the devitrified PFA3 and PFA6 glasses are presented in [47], where we used an innovative Raman imaging technique to present the differences between surface and interior crystallization. The study allowed us to confirm the inhomogeneous character of the glasses and the possible evaporation of P_2_O_5_ out of the surface. The phases identified in this work are compatible with our previous results [47].

It should be also pointed out that if we assume that the crystallizing compounds partially reflect the glass network structure, in the iron-phosphate compounds, only Q^1^ and Q^0^ phosphate structural units exist, whereas, in the case of the aluminum-phosphates, only Q^2^ and Q^0^ are present. Thus, we expect that the aluminum-phosphate glass will be more polymerized than the iron-phosphate. This is in accordance with our previous studies [12], where spectroscopic investigation and ab-initio molecular dynamics simulations showed that aluminum-phosphate glass has a lower number of Q1 structural units and higher Q^2^ and Q^3^ than iron-phosphate glass.

**Figure 13:**
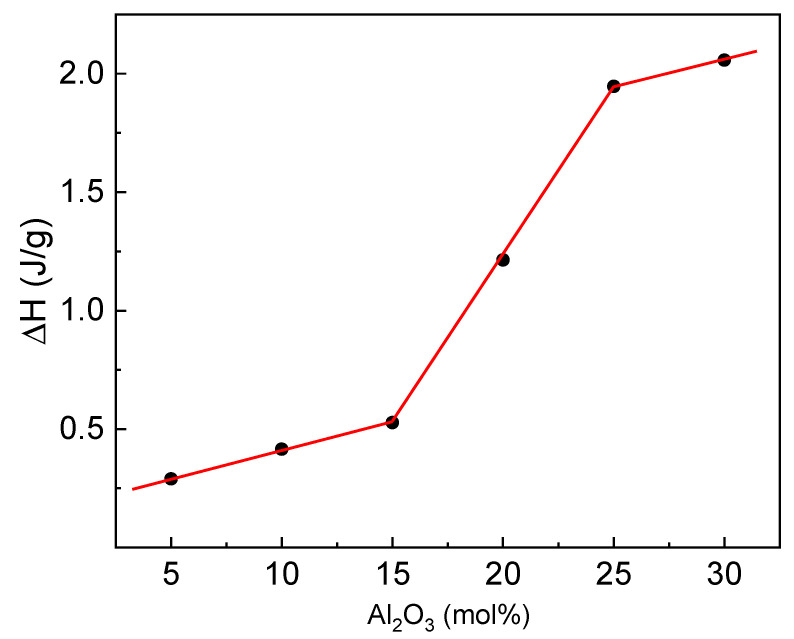
Crystallization enthalpy (ΔH) versus Al_2_O_3_ content.

The dependence of the crystallization enthalpy (ΔH) as a function of the glass composition is presented in Figure 13.

It can be seen that the enthalpy increases with the aluminum content in a different way. Depending on the composition, three specific ranges can be noticed. In the first one, i.e., glasses with low Al_2_O_3_ content, the increase is slower, and the enthalpy is low. In the second one, i.e., glasses with middle aluminum content, the increase is rapid. Lastly, in the third one, i.e., glasses with high aluminum content, the increase becomes slow again, but the enthalpy is much higher. The enthalpy of crystallization can be a measure of the rate of crystal growth [16], which suggests that for the iron-phosphate glasses, the rate is very slow. Nevertheless, according to the XRD studies, the obtained devitrified materials are characterized by a rather high crystallinity degree. The lowest degree is observed for the glasses from the middle range. Thus, the enthalpy in these two ranges should behave oppositely. On the other hand, if we assume that the enthalpy is related to the structural changes during the crystallization, the structure of the iron-phosphate glasses should be closer to the structure of the crystallizing compounds compared to the aluminum-phosphate. Thus, during the crystallization, the network needs to be much more reorganized for the glasses containing the high aluminum content, and their structural features are different than their crystalline counterparts. While the features of the iron-phosphate glasses are similar.

## 4. Conclusions

The influence of the gradual substitution of Fe_2_O_3_ by Al_2_O_3_ on the thermal properties of polyphosphate glasses from the system Fe_2_O_3_-P_2_O_5_ was examined, and its impact on the fundamental technological parameters was discussed.

According to the conducted studies, it can be seen that increasing the aluminum content in the glass leads to an increase in the sintering and transformation temperatures. All of the glasses can be characterized as low-melting. The estimated melting point is below 1000 °C, which is an important observation taking into account the vitrification process. Nevertheless, the addition of aluminum leads to the shortening of the glass, and its viscosity decreases faster with the temperature. This may lead to crystallization of the melt in the case of the high aluminum content glasses. On the other hand, the lower viscosity results in shortening of the homogenization time and thus the reduction of the evaporation of waste constituents. Although Al_2_O_3_ reduces the viscosity of the melt at high temperatures, it impedes the crystallization of iron-phosphate compounds. Thus, it seems that the optimal is almost equal concertation of Al_2_O_3_ and Fe_2_O_3_ in the tested glasses. The composition ensures the relatively low melting temperature, viscosity, and glass crystallization ability. In this way, the partial substitution of Fe_2_O_3_ by Al_2_O_3_ improves the glass properties in terms of waste vitrification, taking into account only thermal properties.

The next important observation is that the simple single DSC measurement can estimate several important technological parameters, such as temperature dependence of glass viscosity, glass forming-ability, and so on.

## Figures and Tables

**Table 1:** Nominal chemical composition of the obtained glasses and that determined by the XRF in brackets (mol%).

Sample	P_2_O_5_	Fe_2_O_3_	Al_2_O_3_
PFA1	70 (69.6)	25 (25.2)	5 (5.2)
PFA2	70 (70.5)	20 (19.2)	10 (10.3)
PFA3	70 (70.2)	15 (14.5)	15 (15.3)
PFA4	70 (69.7)	10 (10.2)	20 (20.1)
PFA5	70 (70.1)	5 (5.2)	25 (24.7)
PFA6	70 (70.4)	0 (0.1)	30 (29.5)

**Table 2:** Characteristic temperatures of the studied glasses.

Sample	T_s_ (°C) ± 10	T_FS_ (°C) ± 10	T_SPH_ (°C) ± 10	T_f_ (°C) ± 10
PFA1	625	770	820	1135
PFA2	635	790	850	1100
PFA3	669	800	870	1170
PFA4	699	820	890	1380
PFA5	731	900	1200	
PFA6	778	930		

**Table 3:** Transformation temperatures (T_g_′, T_g_, T_g_″), the accompanying change in the specific heat (∆C_p_), crystallization temperature (T_c_), crystallization enthalpy (∆H), the activation energy of crystal growth (E_c_), the temperature of the endothermic effect (T_M_), and Angell glass thermal stability parameter (K_A_).

Sample	T_g_′ (°C) ± 2	T_g_ (°C) ± 2	T_g_’’ (°C) ± 2	∆Cp (J/(gK)) ± 2	T_c_ (°C) ± 2	∆H (J/g) ± 5	E_c_ (kJ/mol) ± 5	T_M_ (°C) ± 2	K_A_ (°C) ± 2
PFA1	541	562	577	0.160	847	0.290	544	956	285
PFA2	553	576	590	0.137	845	0.415	565	960	269
PFA3	578	599	615	0.109	841	0.527	586	969	242
PFA4	600	618	633	0.096	842	1.214	628	964	224
PFA5	640	669	664	0.070	854	1.946	448	970	201
PFA6	686	694	700	0.031	786	2.057	356	912	100

## Data Availability

The data presented in this study are available on request from the corresponding author.

## References

[1] Shelby J.E. (2005). Introduction to Glass Science and Technology.

[2] Ojovan I., Lee W.E., Lee W.E. (2010). An Introduction to Nuclear Waste Immobilisation.

[3] Donald I.W. (2010). Waste Immobilization in Glass and Ceramic Based Hosts: Radioactive, Toxic and Hazardous Wastes.

[4] Wang Y., Wang F., Zhou J., Zhu H., Liao Q., Li L., Zhu Y., Yuan Y., Zhang J. (2020). Effect of Molybdenum on Structural Features and Thermal Properties of Iron Phosphate Glasses and Boron-Doped Iron Phosphate Glasses. J. Alloys Compd..

[5] Dube C.L., Stennett M.C., Akhmadaliev S., Hyatt N.C. (2020). Investigation of Ion Irradiation Induced Damages in Iron Phosphate Glasses: Role of Electronic and Nuclear Losses in Glass Network Modification. J. Non-Cryst. Solids.

[6] Joseph K., Stennett M.C., Hyatt N.C., Asuvathraman R., Dube C.L., Gandy A.S., Govindan Kutty K.V., Jolley K., Vasudeva Rao P.R., Smith R. (2017). Iron Phosphate Glasses: Bulk Properties and Atomic Scale Structure. J. Nucl. Mater..

[7] Shi Q., Yue Y., Qu Y., Liu S., Khater G.A., Zhang L., Zhao J., Kang J. (2019). Structure and Chemical Durability of Calcium Iron Phosphate Glasses Doped with La_2_O_3_ and CeO_2_. J. Non-Cryst. Solids.

[8] Bułat K., Sitarz M., Wajda A. (2014). Influence of Aluminium and Boron Ions on the Crystallization of Silicate-Phosphate Glasses from the NaCaPO_4_-SiO_2_ System. J. Non-Cryst. Solids.

[9] Wajda A., Bułat K., Sitarz M. (2016). Structure and Microstructure of the Glasses from NaCaPO_4_–SiO_2_ and NaCaPO_4_–SiO_2_–AlPO_4_ Systems. J. Mol. Struct..

[10] Stoch P., Goj P., Wajda A., Stoch A. (2021). Alternative Insight into Aluminium-Phosphate Glass Network from Ab Initio Molecular Dynamics Simulations. Ceram. Int..

[11] Maslakov K.I., Teterin Y.A., Stefanovsky S.V., Kalmykov S.N., Teterin A.Y., Ivanov K.E. (2017). XPS Study of Uranium-Containing Sodium-Aluminum-Iron-Phosphate Glasses. J. Alloys Compd..

[12] Stoch P., Goj P., Ciecińska M., Jeleń P., Błachowski A., Stoch A., Krakowiak I. (2020). Influence of Aluminum on Structural Properties of Iron-Polyphosphate Glasses. Ceram. Int..

[13] Joseph K., Kutty K.V.G., Goswami M.C., Rao P.R.V. (2014). Viscosity and Crystallization Mechanism of Cesium Loaded Iron Phosphate Glasses. Thermochim. Acta.

[14] Nascimento M.L.F., Souza L.A., Ferreira E.B., Zanotto E.D. (2005). Can Glass Stability Parameters Infer Glass Forming Ability?. J. Non-Cryst. Solids.

[15] Hruby A. (1972). Evaluation of Glass-Forming Tendency by Means of DTA. Czechoslov. J. Phys..

[16] Zheng Q., Zhang Y., Montazerian M., Gulbiten O., Mauro J.C., Zanotto E.D., Yue Y. (2019). Understanding Glass through Differential Scanning Calorimetry. Chem. Rev..

[17] Heide K. (1987). Thermal Analysis of Glass. Thermochim. Acta.

[18] Šesták J. (1996). Use of Phenomenological Kinetics and the Enthalpy versus Temperature Diagram (and Its Derivative—DTA) for a Better Understanding of Transition Processes in Glasses. Thermochim. Acta.

[19] Cabral A.A., Fredericci C., Zanotto E.D. (1997). A Test of the Hrubÿ Parameter to Estimate Glass-Forming Ability. J. Non-Cryst. Solids.

[20] Zheng Q., Mauro J.C. (2017). Viscosity of Glass-Forming Systems. J. Am. Ceram. Soc..

[21] Moynihan C.T. (1993). Correlation between the Width of the Glass Transition Region and the Temperature Dependence of the Viscosity of High-Tg Glasses. J. Am. Ceram. Soc..

[22] Ciecińska M., Goj P., Stoch A., Stoch P. (2019). Thermal Properties of 60P_2_O_5_–(40−x)Al_2_O_3_–xNa_2_O Glasses. J. Therm. Anal. Calorim..

[23] Ciecińska M., Stoch P., Stoch A., Nocuń M. (2015). Thermal Properties of 60P_2_O_5_–20Fe_2_O_3_–20Al_2_O_3_ Glass for Salt Waste Immobilization. J. Therm. Anal. Calorim..

[24] Rincon J.M. (1992). Principles of Nucleation and Controlled Crystallization of Glasses. Polym. Plast. Technol. Eng..

[25] Marotta A., Saiello S., Branda F., Buri A. (1982). Activation Energy for the Crystallization of Glass from DDTA Curves. J. Mater. Sci..

[26] Gražulis S., Chateigner D., Downs R.T., Yokochi A.F.T., Quirós M., Lutterotti L., Manakova E., Butkus J., Moeck P., Le Bail A. (2009). Crystallography Open Database—An Open-Access Collection of Crystal Structures. J. Appl. Crystallogr..

[27] Wajda A., Goldmann W.H., Detsch R., Boccaccini A.R., Sitarz M. (2019). Influence of Zinc Ions on Structure, Bioactivity, Biocompatibility and Antibacterial Potential of Melt-Derived and Gel-Derived Glasses from CaO-SiO_2_ System. J. Non-Cryst. Solids.

[28] Wajda A., Sitarz M. (2018). Structural and Microstructural Comparison of Bioactive Melt-Derived and Gel-Derived Glasses from CaO-SiO_2_ Binary System. Ceram. Int..

[29] Wajda A., Sitarz M. (2016). Structural and Microstructural Studies of Zinc-Doped Glasses from NaCaPO_4_-SiO_2_ System. J. Non-Cryst. Solids.

[30] Wajda A., Goldmann W.H., Detsch R., Grünewald A., Boccaccini A.R., Sitarz M. (2018). Structural Characterization and Evaluation of Antibacterial and Angiogenic Potential of Gallium-Containing Melt-Derived and Gel-Derived Glasses from CaO-SiO_2_ System. Ceram. Int..

[31] Pascual M.J., Duran A., Prado M.O. (2005). A New Method for Determining Fixed Viscosity Points of Glasses. Phys. Chem. Glas..

[32] Callister W.D., David G. (2018). Rethwisch Fundamentals of Materials Science and Engineering.

[33] Ojovan M.I. (2008). Viscosity and Glass Transition in Amorphous Oxides. Adv. Condens. Matter Phys..

[34] Stoch L. (2001). Flexibility of Structure and Glass-Forming Ability: A Chemical Approach. Proceedings of the Glass Physics and Chemistry.

[35] Stoch L., Stoch P. (2012). Significance of Crystallochemical Factors in Chemical Reactions into the Structure of Solids. J.Therm. Anal. Calorim..

[36] Ma L., Brow R.K., Ghussn L., Schlesinger M.E. (2015). Thermal Stability of Na_2_O-FeO-Fe_2_O_3_-P_2_O_5_ Glasses. J. Non-Cryst. Solids.

[37] Kuczek J., Jeleń P., Sułowska J., Szumera M. (2019). Correlation between Glass Transition Effect and Structural Changes in Multicomponent Iron Phosphate-Silicate Glasses. J. Therm. Anal. Calorim..

[38] Stoch P., Goj P., Ciecińska M., Stoch A. (2019). Structural Features of 19Al_2_O_3_-19Fe_2_O_3_-62P_2_O_5_ Glass from a Theoretical and Experimental Point of View. J. Non-Cryst. Solids.

[39] Stoch L., Wacławska I., Środa M. (2004). Thermal Study of the Influence of Chemical Bond Ionicity on the Glass Transformation in (Na_2_O, CaO, MgO)-Al_2_O_3_-SiO_2_ Glasses. J. Therm. Anal. Calorim..

[40] Guo J., Zu F.Q., Chen Z.H., Li X.F., Xi Y., Shen R.R., Zhang Y. (2006). Attempt to Depict Glass Forming Ability of Bulk Metallic Glasses Using the Criterion of the Total Relaxation Time at the Glass Transition. J. Non-Cryst. Solids.

[41] Soliman A.A., Kashif I. (2010). Copper Oxide Content Dependence of Crystallization Behavior, Glass Forming Ability, Glass Stability and Fragility of Lithium Borate Glasses. Phys. B Condens. Matter.

[42] Szumera M., Waclawska I. (2012). Effect of Molybdenum Addition on the Thermal Properties of Silicate–Phosphate Glasses. J. Therm. Anal. Calorim..

[43] Kodama M., Kojima S. (2002). Anharmonicity and Fragility in Lithium Borate Glasses. J. Therm. Anal. Calorim..

[44] Vilgis T.A. (1993). Strong and Fragile Glasses: A Powerful Classification and Its Consequences. Phys. Rev. B.

[45] Elbouaanani L.K., Malaman B., Gérardin R., Ijjaali M. (2002). Crystal Structure Refinement and Magnetic Properties of Fe_4_(P_2_O_7_)_3_ Studied by Neutron Diffraction and Mössbauer Techniques. J. Solid State Chem..

[46] Ray C.S., Fang X., Karabulut M., Marasinghe G.K., Day D.E. (1999). Effect of Melting Temperature and Time on Iron Valence and Crystallization of Iron Phosphate Glasses. J. Non-Cryst. Solids.

[47] Goj P., Wajda A., Stoch P. (2021). Raman Imaging as a Useful Tool to Describe Crystallization of Aluminum/Iron-Containing Polyphosphate Glasses. J. Eur. Ceram. Soc..

